# Fetuin-A as a Potential Biomarker of Metabolic Variability Following 60 Days of Bed Rest

**DOI:** 10.3389/fphys.2020.573581

**Published:** 2020-10-19

**Authors:** Kiera Ward, Edwin Mulder, Petra Frings-Meuthen, Donal J. O’Gorman, Diane Cooper

**Affiliations:** ^1^Faculty of Science and Health, Athlone Institute of Technology, Athlone, Ireland; ^2^Department of Muscle and Bone Metabolism, Institute of Aerospace Medicine, German Aerospace Center (Deutsches Zentrum für Luft- und Raumfahrt, DLR), Cologne, Germany; ^3^3U Diabetes Partnership, School of Health and Human Performance, Dublin City University, Dublin, Ireland; ^4^National Institute for Cellular Biotechnology, Dublin City University, Dublin, Ireland

**Keywords:** bed rest, fetuin-A, hepatokine, insulin sensitivity, liver, metabolism

## Abstract

**Background**: Fetuin-A is a hepatokine linked to the development of insulin resistance. The purpose of this study was to determine if 60 days head-down-tilt (HDT) bed rest increased circulating fetuin-A and if it was linked to whole body insulin sensitivity (IS). Additionally, we examined whether reactive jump training (RJT) could alleviate the metabolic changes associated with bed rest.

**Methods**: 23 young men (29 ± 6 years, 181 ± 6 cm, 77 ± 7 kg) were randomized to a control (CTRL, *n* = 11) or RJT group (JUMP, *n* = 12) and exposed to 60 days of bed rest. Before and after bed rest, body composition and $\dot{\text{V}}\text{O}{}_{2\,\text{p}\,\text{e}\,\text{a}\,\text{k}}$ were measured and an oral glucose tolerance test was performed to estimate IS. Circulating lipids and fetuin-A were measured in fasting serum.

**Results**: Body weight, lean mass, and $\dot{\text{V}}\text{O}{}_{2\,\text{p}\,\text{e}\,\text{a}\,\text{k}}$ decreased in both groups following bed rest, with greater reductions in CTRL (*p* < 0.05). There was a main effect of time, but not the RJT intervention, for the increase in fetuin-A, triglycerides (TG), area under the curve for glucose (AUCG) and insulin (AUCI), and the decrease in Matsuda and tissue-specific IS (*p* < 0.05). Fetuin-A increased in participants who became less insulin sensitive (*p* = 0.019). In this subgroup, liver IS and adipose IS decreased (*p* < 0.05), while muscle IS was unchanged. In a subgroup, where IS did not decrease, fetuin-A did not change. Liver IS increased (*p* = 0.012), while muscle and adipose tissue IS remained unchanged.

**Conclusions**: In this study, we report an increase in circulating fetuin-A following 60 days of bed rest, concomitant with reduced IS, which could not be mitigated by RJT. The amount of fetuin-A released from the liver may be an important determinant of changes in whole body IS. In this regard, it may also be a useful biomarker of individual variation due to inactivity or lifestyle interventions.

## Introduction

Physical inactivity and exposure to microgravity induce aging-like phenotypic changes that are associated with the etiology of many chronic diseases (Bergouignan et al., 2011; Hart and Zernicke, 2020). The physiological changes in space and during head-down-tilt (HDT) bed rest, the Earth-based analogue of microgravity, have been well-described and include altered cardiovascular capacity, bone loss, muscle atrophy, impaired functional capacity, and metabolic dysregulation, among others (Bergouignan et al., 2011; Narici and de Boer, 2011; Ade et al., 2015; Vico and Hargens, 2018; Konda et al., 2019). There is evidence that underlying mechanisms, such as insulin resistance, play an important role in the regulation of whole body metabolic changes (Gratas-Delamarche et al., 2014). Insulin resistance is a multi-faceted disruption of the action of insulin in skeletal muscle, adipose tissue, vasculature, brain, and the liver, leading to hyperinsulinemia and reduced glucose disposal (Cox-York and Pereira, 2020). It is also associated with impaired oxidative capacity, increased circulating and deposition of lipids, and metabolic inflexibility. These whole body and cellular changes have been observed following bed rest studies, even when energy balance is maintained (Bergouignan et al., 2006, 2009, 2011; Kenny et al., 2017; Rudwill et al., 2018). The severity of insulin resistance has been found to vary considerably between individuals and between the key target organs (Unnikrishnan, 2004; Abdul-Ghani et al., 2007). Therefore, understanding the individual variability in insulin resistance could provide personalized information on disease etiology and individualized interventions to maintain health.

A key strategy for monitoring metabolic homeostasis is communication between peripheral tissues *via* secreted proteins, which perform autocrine, paracrine, and endocrine actions. Disruption of protein production and target-tissue action underpin the development of metabolic dysfunction including insulin resistance (Priest and Tontonoz, 2019). The quantitative measurement of circulating protein biomarkers is a relatively easy and minimally-invasive means of identifying and understanding the etiology of insulin resistance.

The liver is a major regulator of systemic glucose metabolism. Liver-derived proteins, known as hepatokines, are released into circulation and some of them are known to enhance or attenuate insulin sensitivity (Stefan and Häring, 2013; Iroz et al., 2015; Choi, 2016). Fetuin-A is a novel hepatokine, encoded by the alpha-2-HS-glycoprotein (AHSG) gene in humans and is a known regulator of metabolism (Stefan and Häring, 2013). In particular, an increase in fetuin-A is associated with the development of insulin resistance and the pathophysiology of type 2 diabetes mellitus (T2DM). Fetuin-A impairs the insulin signaling cascade by binding to the tandem fibronectin type 3 domains present on the extracellular portion of the transmembrane β-subunit of the insulin receptor, attenuating tyrosine kinase signaling and leading to reduced glucose uptake (Goustin et al., 2013; Ochieng et al., 2018). Additionally, fetuin-A has been proposed to inhibit the production of the insulin-sensitizing hormone adiponectin in adipocytes, indirectly leading to a decrease in insulin sensitivity (Hennige et al., 2008). Finally, fetuin-A is implicated in lipid-induced insulin resistance by acting as an intermediary between palmitate and toll-like receptor 4 (TLR4) leading to adipose tissue inflammation and insulin resistance (Pal et al., 2012).

Exercise training, using a wide-range of modalities, is known to promote glucose control and improve insulin sensitivity in healthy and clinical populations (Ennequin et al., 2019). One of the mechanisms that contributes to improved metabolic health is training-induced alterations in the production and secretion of pro-inflammatory and anti-inflammatory cytokines from tissues of metabolic importance. Numerous studies have reported that exercise training decreases the secretion of fetuin-A from the liver concomitant with improvements in whole body and liver insulin sensitivity in patients with metabolic disease (Malin et al., 2013, 2014; Lee et al., 2017; Ennequin et al., 2019).

The purpose of this study was to determine if 60 days of extreme physical inactivity increased circulating fetuin-A and if those changes correlated with whole body insulin sensitivity. We used the European Space Agency 60 day bed rest model, where subjects were maintained in energy balance, despite a decrease in physical activity. This unique approach means fat accumulation is not a confounding factor on the metabolic outcomes. In addition, we examined whether reactive jump training (RJT), a countermeasure used to maintain skeletal muscle mass, was able to attenuate the deterioration in metabolic health that occurs with prolonged inactivity.

## Materials and Methods

### General Study Information

This research was conducted as part of the “Reactive jumps in a sledge jump system as a countermeasure during long-term bed rest” (RSL) study funded by the European Space Agency, which ran as two separate bed rest campaigns, commencing in August 2015 and January 2016, respectively. This parallel-design randomized controlled training study was conducted at the “envihab” facility at the German Aerospace Center (DLR). A detailed description of the subject recruitment procedures, experimental conditions, diet, countermeasure, and training protocol have been published previously (Kramer et al., 2017b).

In brief, the study was split into three phases: a 15-day baseline data collection phase (BDC-15 to BDC-1), 60 days of strict 6° head-down-tilt bed rest (HDT1 to HDT60), followed by a post-intervention testing phase (R + 0 to R + 14), with a total duration of 90 days. Subjects were randomly assigned to a control group (CTRL) or intervention group involving reactive jump exercise (JUMP). For the duration of the bed rest period, subjects remained at the 6° HDT angle for 24 h/day.

The inclusion criteria have been described previously (Kramer et al., 2017b) but included men, aged 20–45 years, body mass index (BMI) 20–26 kg/m^2^, non-smoker, no medication, no history of bone fractures, non-competitive athlete, and no medical conditions. Initially, 24 male volunteers were enrolled for the study, however one subject discontinued during baseline data collection due to medical reasons unrelated to the study. In addition, two subjects were ambulated after 49-days HDT (CTRL) and 50-days HDT (JUMP) due to medical reasons but all post-bed rest data were collected except for $\dot{\text{V}}\text{O}{}_{2\,\text{p}\,\text{e}\,\text{a}\,\text{k}}$.

On HDT1, participants were randomly assigned to either the control group (CTRL, *n* = 11, age 28 ± 6 years, BMI 23.3 ± 2 kg/m^2^) or the countermeasure group (JUMP, *n* = 12, age 30 ± 7 years, BMI 23.8 ± 2 kg/m^2^), which performed RJT 5–6 days per week in a horizontal sledge jump system. A total of 48 training sessions were completed during the HDT bed rest period. Each training session involved a varying amount of repetitive hops and countermovement jumps with an average load equal to or exceeding 80% of the individual’s body weight. The maximal workload in one session excluding breaks did not exceed 4 min. During the entire study, the subjects received a strictly controlled and individualized diet, which was tailored to maintain energy balance. The study protocols were approved by the Ethics Committee of the North Rhine Medical Association (Ärztekammer Nordrhein) in Düsseldorf, Germany, as well as the Federal Office for Radiation Protection (Bundesamt für Strahlenschutz). All subjects gave their written informed consent before commencing the study in accordance with the Declaration of Helsinki. This study was registered with the German Clinical Trial Registry (#DRKS00012946, 18th September 2017).

### Body Weight and Body Composition

Measurements of body weight and body composition were taken on numerous days before and after bed rest. This is a large scale bed rest study and data are reported by different research groups. The core data have been published elsewhere (Kramer et al., 2017b), however, this study has compared measurements of body weight and body composition recorded on BDC-3 and HDT60. Body weight was measured daily following the first urine void of the day (DVM 5703, Sartorius, Göttingen, Germany). Body composition was examined with dual-energy X-ray absorptiometry (DEXA), using the whole body scan feature on the Prodigy Full Pro (GE Healthcare GmbH, Solingen, Germany) and the manufacturer’s enCORE software (version 16.10.151) was used to generate automated reports of total lean mass, fat mass, and bone mineral content.

### Peak Oxygen Consumption Test

Peak aerobic capacity ($\dot{\text{V}}\text{O}{}_{2\,\text{p}\,\text{e}\,\text{a}\,\text{k}}$) was measured on BDC-8 and R + 1 using cycle ergometry (Lode, Groningen, The Netherlands) as previously described (Kramer et al., 2017a). In brief, after an initial 5 min of seated rest, subjects were instructed to start pedaling and to maintain a cadence of 75 revolutions per minute (rpm). The warm-up consisted of 3 min cycling at 50 W, followed by 1 min stages, in which the load was increased by 25 W per stage until volitional exhaustion, despite strong verbal encouragement. If the peak respiratory exchange ratio (RER) did not exceed 1.10, the trial was deemed not exhaustive and not considered for further analyses. Due to the absence of post-bed rest data, two subjects were removed from our analysis of $\dot{\text{V}}\text{O}{}_{2\,\text{p}\,\text{e}\,\text{a}\,\text{k}}$.

### Oral Glucose Tolerance Test

An oral glucose tolerance test (OGTT) was performed after a 12-h overnight fast, in the morning of BDC-5 and HDT59. A catheter was placed in the antecubital vein and blood samples were drawn before and at 30 min intervals (30, 60, 90, and 120 min) after the ingestion of a 75 g glucose equivalent (ACCU Chek® Dextro OGT, Roche Diagnostics Deutschland GmbH, Mannheim) dissolved in 300 ml water. Following sample extraction, serum was left to coagulate at room temperature for 30 min before centrifugation, while vacutainers containing fluoride and EDTA were centrifuged immediately (184 × *g*, 4°C, 10 min). Serum and plasma were then aliquoted and stored at −80°C until analysis.

### Plasma Volume Correction

As the 6° HDT angle induces a fluid shift and consequent loss of plasma volume (PV), the change in PV was calculated and the concentrations of biochemical parameters following HDT bed rest were corrected for changes in hemoconcentration (Dill and Costill, 1974; Alis et al., 2016). The change in PV (Δ%PV) was calculated as follows: Δ%PV = 100^*^((Hb_pre_/Hb_post_)^*^(100–Htc_post_)/(100–Htc_pre_)–1), where hemoglobin (Hb) is given in g/dL and hematocrit (Hct) is expressed as a percentage (%). To correct measured parameters for changes in PV, the following calculation was used: [parameter]c = [parameter]u*(1 + ΔPV(%)/100), where the *c* and *u* indices represent corrected and uncorrected concentrations, respectively.

### Biochemical Analysis and Assays

Concentrations of glucose, non-esterified fatty acids (NEFA), total cholesterol, LDL-cholesterol (LDL), HDL-cholesterol (HDL), and triglycerides (TG) were measured using colorimetric assay kits on the Randox RX Daytona™ (Crumlin, United Kingdom). Serum insulin was quantified using an immunoassay method on the Cobas® 8000 modular analyser (module e602, Roche Diagnostics, North America). Area under the curve for glucose (AUCG) and insulin (AUCI) were calculated according to the trapezoidal rule. Indexes of insulin resistance and insulin sensitivity including the Matsuda index, liver insulin sensitivity (liver IS), muscle insulin sensitivity (muscle IS), and adipose tissue insulin resistance (adipose IR) were calculated using previously reported equations (Supplementary Material; Matsuda and DeFronzo, 1999; Abdul-Ghani et al., 2007; Lomonaco et al., 2012). Using fasting serum samples from the OGTT, concentrations of fetuin-A were assayed in duplicate using the human fetuin-A quantikine ELISA kit, according to the manufacturer’s instructions (Cat No: DFTA00. R&D Systems Inc. Minneapolis, United States). The intra-assay coefficient of variance (%CV) was 11%.

### Statistical Analysis

All experimental data are presented as mean ± SD. Normality of distribution for each variable was evaluated using the Shapiro-Wilk test and data violating the assumption of normality was transformed. Differences in baseline characteristics between the two experimental groups were assessed using independent samples *t*-tests or the non-parametric Mann-Whitney U test. Physical and metabolic changes in response to bed rest were analyzed using a mixed between-within factorial analysis of variance using time as the within-group factor and experimental group as the between-group factor (CTRL and JUMP). When a statistically significant interaction was found, simple main effects are reported as mean (M) and standard error (SE). Statistical analysis was performed in SPSS 26.0 (IBM Corp., Armonk, NY, United States) considering a two-sided 0.05 significance level.

### In-Depth Data Analysis

An association between insulin sensitivity and fetuin-A has been well-established within the literature (Ochieng et al., 2018; Bourebaba and Marycz, 2019). In the current study, pre- to post-differences in insulin sensitivity and fetuin-A exhibited significant effects of time only, so to further explore the relationship between both variables additional subanalysis was conducted. The participant data from the two experimental groups were pooled and then divided into two subgroups based on participants who improved (↑ Matsuda, *n* = 6, CTRL *n* = 4, and JUMP *n* = 2) or reduced (↓ Matsuda, *n* = 17, CTRL *n* = 7, and JUMP *n* = 10) insulin sensitivity post-HDT bed rest. Paired sample *t*-tests were used to assess the significance of pre- to post-bed rest changes in physical and metabolic parameters in both subgroups.

## Results

### Physical Characteristics: Body Weight, Body Composition, and $\dot{\text{V}}\text{O}{}_{2\,\text{p}\,\text{e}\,\text{a}\,\text{k}}$

Anthropometric measurements were obtained on BDC-3 and HDT60 (Table 1). No significant between-group differences in physical characteristics were identified at baseline. Following 60 days of HDT bed rest, body weight decreased significantly in both groups, with a larger reduction observed in the CTRL group (*M* = −3.63 kg, *SE* = 0.54 kg, *p* < 0.001) compared to the JUMP group (*M* = −2.23 kg, *SE* = 0.28 kg, *p* < 0.001). Similarly, bed rest significantly reduced lean mass, with a higher decline noticeable in the CTRL group (*M* = −3.91 kg, *SE* = 0.70 kg, *p* < 0.001) in comparison to the JUMP group (*M* = −1.34 kg, *SE* = 0.32 kg, *p* = 0.002). Fat mass decreased significantly in the JUMP group (*M* = −0.87 kg, *SE* = 0.23 kg, *p* = 0.003) but did not change significantly in the CTRL group (*M* = 0.10 kg, *SE* = 0.31 kg, *p* = 0.757) following HDT bed rest. Bone mineral content did not change significantly. Absolute $\dot{\text{V}}\text{O}{}_{2\,\text{p}\,\text{e}\,\text{a}\,\text{k}}$, measured on BDC-8 and R + 1, decreased significantly in both groups after HDT bed rest, with a greater loss identified in the CTRL group (*M* = −1.28 L/min, *SE* = 0.17 L/min, *p* < 0.001) in comparison to the JUMP group (*M* = −0.33 L/min, *SE* = 0.15 L/min, *p* = 0.049). $\dot{\text{V}}\text{O}{}_{2\,\text{p}\,\text{e}\,\text{a}\,\text{k}}$, when normalized for changes in lean mass, decreased significantly in the CTRL group (*M* = −18.82 ml/kgLW/min, *SE* = 2.26 ml/kgLM/min, *p* < 0.001) but did not change in the JUMP group (*M* = −4.58, *SE* = 2.17, *p* = 0.063) after HDT bed rest.

**Table 1:** Effects of 60 days HDT bed rest on measures of anthropometry and cardiorespiratory capacity.

Measurement	CTRL (*n* = 11)		JUMP (*n* = 12)		Statistics		
	Pre	Post	Pre	Post	Time	Int	T^*^Int
Age (years)	28 ± 6		30 ± 7				
Height (cm)	181 ± 5		181 ± 7				
BMI (kg/m^2^)	23.33 ± 2.03	22.22 ± 1.67^*^	23.75 ± 1.80	23.07 ± 1.81^*^	<**0.001**	0.410	**0.021**
BW (kg)	76.10 ± 8.06	72.47 ± 6.76^*^	77.85 ± 6.55	75.63 ± 6.39^*^	<**0.001**	0.405	**0.027**
LM (kg)	56.94 ± 6.57	53.03 ± 5.11^*^	56.41 ± 5.18	55.08 ± 4.29^*^	<**0.001**	0.731	**0.002**
FM (kg)	16.91 ± 3.95	17.00 ± 3.41	19.21 ± 6.42	18.34 ± 6.18^*^	0.055	0.412	**0.018**
BMC (kg)	3.14 ± 0.36	3.14 ± 0.37	3.00 ± 0.32	3.00 ± 0.32	0.605	0.355	0.800
**Measurement**	**CTRL (*n*** = **10)**		**JUMP (*n*** = **10)**		**Statistics**		
	**Pre**	**Post**	**Pre**	**Post**	**Time**	**Int**	T^*^Int
$\dot{\text{V}}\text{O}{}_{2\,\text{p}\,\text{e}\,\text{a}\,\text{k}}$ (L/min)	3.85 ± 0.68	2.57 ± 0.48^*^	3.32 ± 0.76	2.99 ± 0.53^*^	<**0.001**	0.848	**0.001**
$\dot{\text{V}}\text{O}{}_{2\,\text{p}\,\text{e}\,\text{a}\,\text{k}}$ (ml/kgLM/min)	67.55 ± 8.42	48.74 ± 9.35^*^	58.72 ± 10.95	54.14 ± 8.03	<**0.001**	0.660	<**0.001**

Data are presented as mean ± standard deviation (SD). Significant *p* < 0.05 are indicated in bold. Anthropometric measurements were taken on BDC-3 and HDT60. $\dot{\text{V}}\text{O}{}_{2\,\text{p}\,\text{e}\,\text{a}\,\text{k}}$ was measured on BDC-8 and R + 1. When a significant interaction effect was found, an asterisk (^*^) denotes a significant difference from pre in each intervention group. CTRL, control group; JUMP, jumping countermeasure group; Time, main effect of time; Int, main effect of intervention; T^*^Int, time^*^intervention interaction effect; BMI, body mass index; BW, body weight; LM, lean mass; FM, fat mass; BMC, bone mineral content; $\dot{\text{V}}\text{O}{}_{2\,\text{p}\,\text{e}\,\text{a}\,\text{k}}$, peak aerobic capacity.

### Metabolic Characteristics: Glucose Tolerance, Insulin Sensitivity, Lipid Metabolism, and Fetuin-A

**Figure 1:**
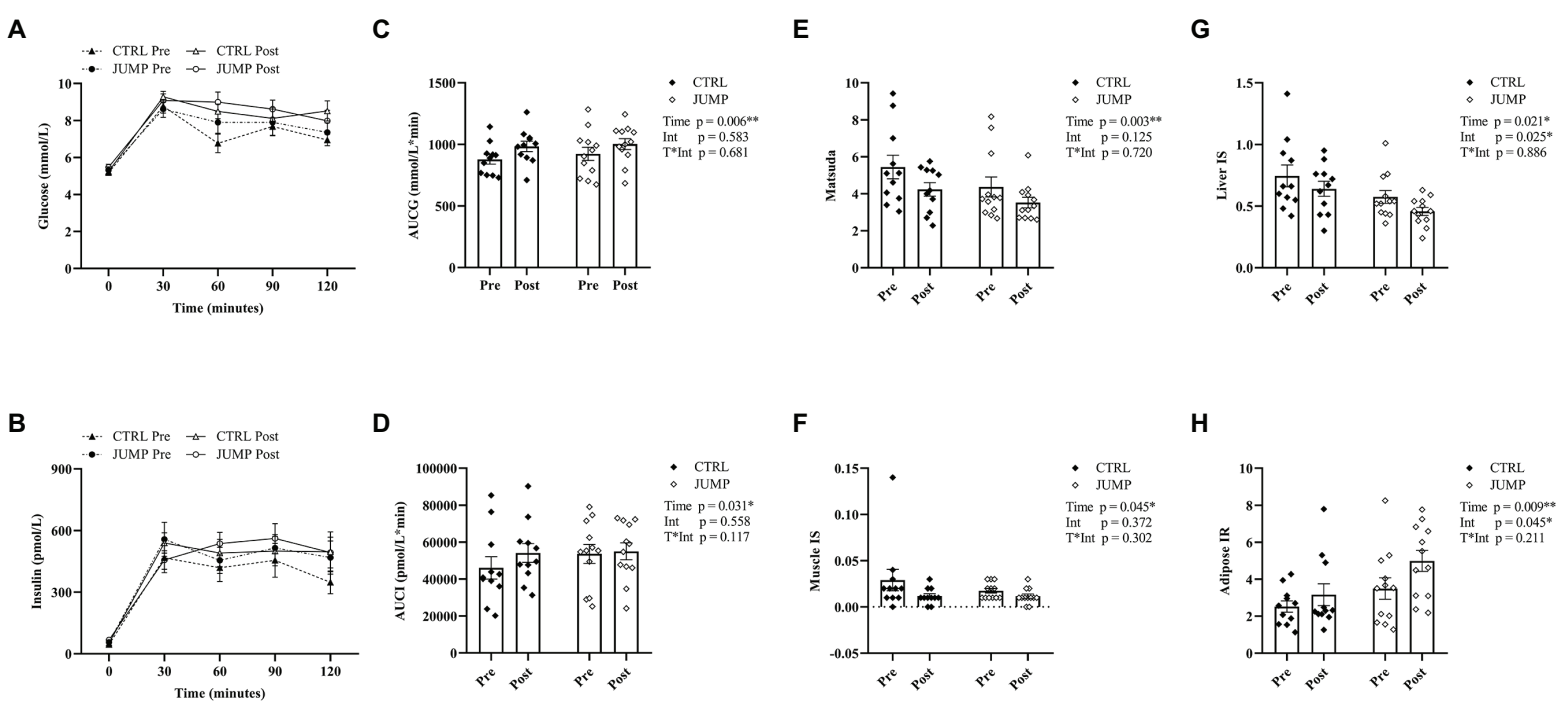
The effects of 60 days of HDT bed rest on metabolic variables measured on BDC-5 (pre) and HDT59 (post). Data are presented as mean ± standard error of mean (SEM). The glucose and insulin response curve to the OGTT are displayed in **(A)** and **(B)**, respectively. The 120 min area under the curve totals for glucose are shown in **(C)** and for insulin in **(D)**. The pre to post changes in OGTT derived indexes of Matsuda, liver IS, muscle IS, and adipose IR are presented in **(E**–**H)**. Abbreviations: CTRL, control group; JUMP, jumping countermeasure group; AUCG, area under the curve for glucose for 120 min; AUCI, area under the curve for insulin for 120 min; IS, insulin sensitivity; IR, insulin resistance; Time, main effect of time; Int, main effect of intervention; T^*^Int, time^*^intervention interaction effect. ^*^p ≤ 0.05 and ^**^p ≤ 0.010.

**Figure 2:**
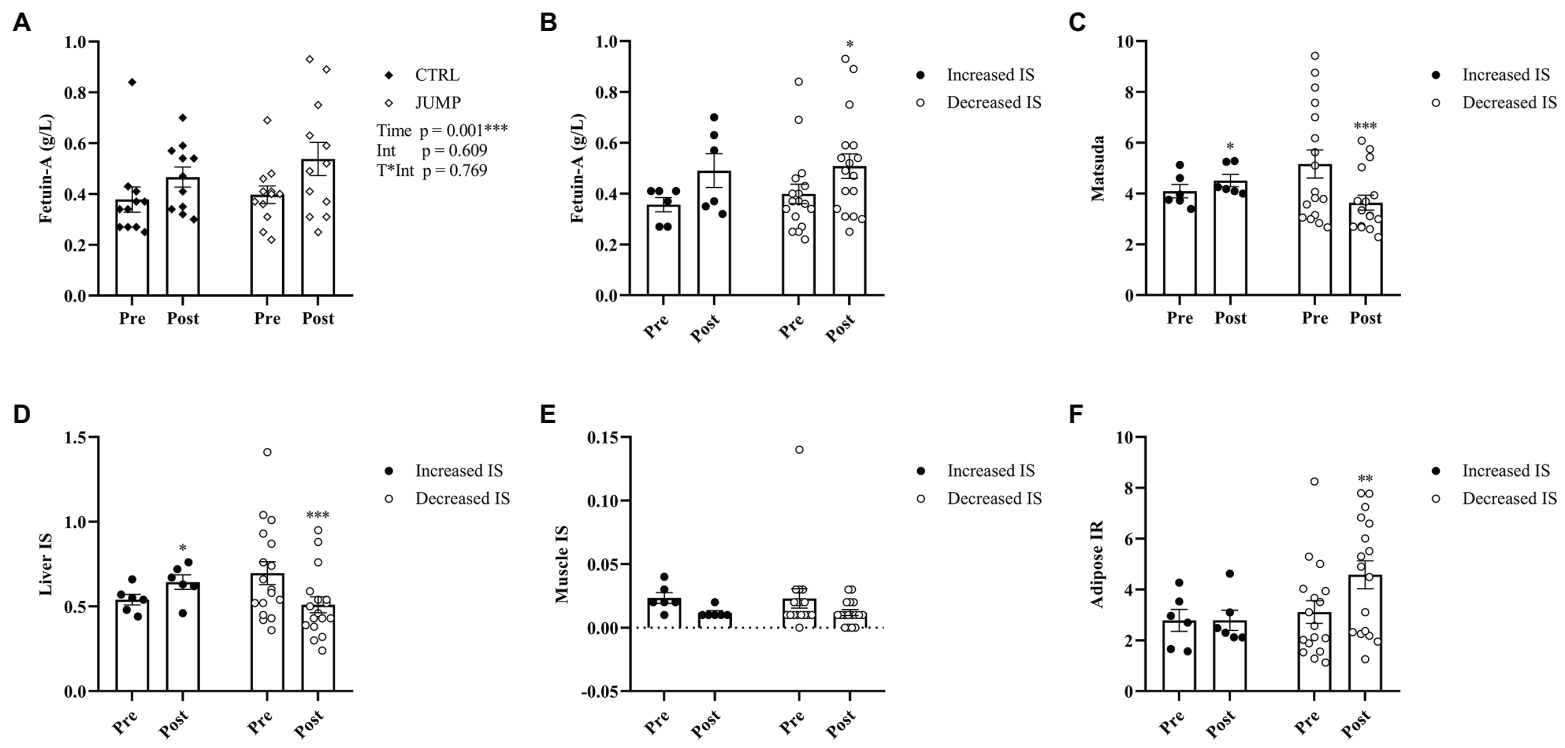
**(A)** Circulating fetuin-A in the CTRL and JUMP groups before and after 60 days of HDT bed rest. **(B**–**F)** The effect of HDT bed rest on fetuin-A and OGTT derived indexes of insulin sensitivity and insulin resistance when subjects were divided into two subgroups based on an increase (*n* = 6) or decrease (*n* = 17) in insulin sensitivity (Matsuda) post-HDT bed rest. Data are presented as mean ± SEM. Abbreviations: CTRL, control group; JUMP, jumping countermeasure group; IS, insulin sensitivity; IR, insulin resistance; Time, main effect of time; Int, main effect of intervention; T^*^Int, time^*^intervention interaction effect. ^*^p ≤ 0.05, ^**^p ≤ 0.010, and ^***^p ≤ 0.001.

The changes in metabolic parameters are presented in Figure 1 and Table 2. No significant between-group differences in metabolic characteristics were found at baseline. Metabolic characteristics were corrected for changes in hemoconcentration following HDT bed rest (mean ∆PV% CTRL 0.88%, JUMP -2.13%). NEFA, total cholesterol, fasting glucose (glucose_0_), and 2-h insulin (insulin_120_) did not change significantly following HDT bed rest. There was a significant effect of time, but not intervention, for the increase in 2-h glucose (glucose_120_; *p* = 0.010), TG (*p* = 0.013), and LDL (*p* = 0.004) and decrease in HDL (*p* < 0.001) after HDT bed rest (Table 2). A significant effect of time and intervention was identified for fasting insulin (insulin_0_), which increased significantly following HDT bed rest and was significantly higher overall in the JUMP group (Table 2). There was a significant effect of time, but not intervention, for the increase in AUCG and AUCI post-HDT bed rest (Figures 1A–D). A significant main effect of time only was also identified for the decrease in Matsuda and muscle IS following HDT bed rest (Figures 1E–F). The main effect of time and intervention were statistically significant for the change in liver IS and adipose IR following HDT bed rest (Figures 1G–H). Liver IS decreased significantly after HDT bed rest and was significantly higher overall in the CTRL group. In contrast, adipose IR increased significantly following HDT bed rest and was found to be significantly higher overall in the JUMP group. A significant main effect of time, but not intervention, was found for the increase in circulating fetuin-A (Figure 2A). Fetuin-A increased from 0.38 ± 0.16 to 0.47 ± 0.13 g/L in the CTRL group and from 0.40 ± 0.12 to 0.54 ± 0.23 g/L in the JUMP group after HDT bed rest.

**Table 2:** The effects of 60 days HDT bed rest on metabolic characteristics.

Measurement	CTRL (*n* = 11)		JUMP (*n* = 12)		Statistics		
	Pre	Post	Pre	Post	Time	Int	T^*^Int
Glucose_0_ (mmol/L)	5.18 ± 0.40	5.24 ± 0.51	5.32 ± 0.64	5.47 ± 0.59	0.447	0.311	0.732
Glucose_120_ (mmol/L)	6.95 ± 1.01	8.51 ± 1.80	7.36 ± 1.82	7.98 ± 1.98	**0.010**	0.919	0.234
Insulin_0_ (pmol/L)	45.00 ± 13.09	51.48 ± 16.49	55.55 ± 14.92	67.22 ± 19.14	**0.012**	**0.036**	0.437
Insulin_120_^†^ (pmol/L)	347.16 ± 184.12	496.38 ± 321.36	468.39 ± 281.21	492.68 ± 260.41	0.054	0.587	0.264
NEFA (mmol/L)	0.40 ± 0.13	0.41 ± 0.16	0.41 ± 0.14	0.52 ± 0.17	0.124	0.232	0.215
TG (mmol/L)	0.87 ± 0.26	1.00 ± 0.26	1.16 ± 0.45	1.21 ± 0.41	**0.013**	0.102	0.245
CHOL (mmol/L)	4.09 ± 0.72	4.27 ± 0.86	4.11 ± 0.57	4.06 ± 0.57	0.619	0.710	0.381
HDL (mmol/L)	1.16 ± 0.17	0.99 ± 0.19	1.08 ± 0.27	0.91 ± 0.17	<**0.001**	0.304	0.955
LDL (mmol/L)	2.76 ± 0.71	3.10 ± 0.71	2.73 ± 0.51	2.99 ± 0.56	**0.004**	0.780	0.647

Data are presented as mean ± standard deviation (SD). Significant *p* < 0.05 are indicated in bold. ^†^Denotes that data were transformed for statistical analysis. Metabolic characteristics were measured on BDC-5 and HDT59. CTRL, control group; JUMP, jumping countermeasure group; Time, main effect of time; Int, main effect of intervention; T^*^Int, time^*^intervention interaction effect; Glucose_0_, fasting glucose; Glucose_120_, glucose concentrations 120 min after the glucose load; Insulin_0_, fasting insulin; Insulin_120_, insulin concentrations 120 min after the glucose load; NEFA, non-esterified fatty acids; TG, triglycerides; CHOL, total cholesterol; HDL, high-density lipoprotein cholesterol; LDL, low-density lipoprotein cholesterol.

### Subanalysis Exploring the Relationship Between Insulin Sensitivity and Fetuin-A

Changes in the physical and metabolic parameters in subgroups with decreased and increased insulin sensitivity following HDT bed rest are presented in Figures 2B–F and Supplementary Tables 1 and 2.

In the subgroup with improved insulin sensitivity following HDT bed rest, the pre- to post-increase in liver IS was statistically significant. Despite this, muscle IS and adipose IR did not change significantly after HDT bed rest. In subjects who became more insulin-sensitive following HDT bed rest, the change in fetuin-A was not statistically significant.

In the opposing subgroup, with reduced insulin sensitivity after HDT bed rest, the pre- to post-decrease in liver IS was statistically significant. Adipose IR increased significantly following HDT bed rest. The change in muscle IS was not statistically significant. Interestingly, in those who became less insulin-sensitive during HDT bed rest, circulating concentrations of fetuin-A significantly increased.

## Discussion

The main findings of the current study demonstrate that 60 days of HDT bed rest elicited a significant increase in fetuin-A concomitant with reduced insulin sensitivity, which could not be mitigated by RJT. As considerable individual differences have been found in the responsiveness to lifestyle interventions, we compared changes in metabolic variables in subgroups with decreased and increased insulin sensitivity following HDT bed rest. Our results suggest that fetuin-A may have a role in the regulation of peripheral insulin sensitivity during bed rest and physical inactivity. In addition, fetuin-A has potential as a biomarker to track individual changes in metabolic homeostasis.

Exercise and diet countermeasures have been widely implemented to abrogate deconditioning during bed rest. The RJT protocol combined plyometric movements with high rates of force development with the aim of preserving musculoskeletal mass and strength (Kramer et al., 2017a). The results, from previous publications, show that this time-efficient countermeasure attenuated the loss of whole body lean mass, leg lean mass, $\dot{\text{V}}\text{O}{}_{2\,\text{p}\,\text{e}\,\text{a}\,\text{k}}$ (Kramer et al., 2017a,b), and myofiber size and phenotype (Blottner et al., 2019). While RJT had protective effects for muscle function, it could not prevent the dysregulation of glucose and lipid metabolism. Similar results were reported following flywheel exercise (Bergouignan et al., 2006) and the authors, in this case, suggested that insufficient energy expenditure during the training sessions may be the key factor. While the RJT is a form of high intensity interval training consisting of 48 jumps and 30 hops, the overall workload may not be sufficient to improve insulin sensitivity. There was a decrease in fat mass (0.9 kg), indicating a negative energy balance in the JUMP group but this may be due to the challenges of estimating energy expenditure of the exercise protocol.

Another challenge for countermeasure design and implementation is the individual variation in response to lifestyle interventions (Bouchard and Rankinen, 2001; Solomon, 2018; O’Donoghue et al., 2019). While a specific exercise program may, on average, be effective there can be a broad range in the individual response. This is particularly important for long-term missions in microgravity, where a standard countermeasure program may not confer the same benefit to all the astronauts. It will be important to have individualized countermeasure programs that can be monitored and adjusted depending on the response. While changes in muscle mass can be observed with relative ease, monitoring changes in metabolism is more challenging in microgravity. The role of circulating biomarkers may serve as a simple and effective strategy to track metabolic changes on the health continuum and guide countermeasure recommendations. Previous research has reported that the beneficial and detrimental effects of physical activity and inactivity, respectively, are linked with changes in circulating biomarkers, which are key messengers for inter-organ communication (Pedersen and Febbraio, 2012; Ennequin et al., 2019; Mika et al., 2019).

Fetuin-A is a multifunctional glycoprotein that is predominately synthesized and secreted by hepatocytes (Bourebaba and Marycz, 2019) and associated with insulin action (Ochieng et al., 2018). To the best of our knowledge, fetuin-A has not been previously reported following bed rest and we report a significant increase in circulating fetuin-A following 60 days of HDT bed rest, irrespective of the experimental group. The regulation of hepatic fetuin-A secretion is incompletely understood (Haukeland et al., 2012). Previous research has reported that fetuin-A mRNA expression in the liver correlated positively with hepatic triglyceride content and homeostatic model of insulin resistance (HOMA-IR) and these associations remained significant after adjustment for BMI (Peter et al., 2018). These findings were supported by a cross-sectional study reporting higher fetuin-A in subjects with elevated liver fat content (Stefan et al., 2006). Analysis of longitudinal data following a lifestyle intervention found that the changes in fetuin-A paralleled the changes in liver fat (Stefan et al., 2006). Collectively, this evidence presents fetuin-A as a possible biological and predictive marker of metabolic disease.

Fetuin-A is also responsive to exercise training and a number of studies have reported a decrease in circulating levels with accompanying improvements in body composition, liver fat, and insulin sensitivity (Malin et al., 2013, 2014). Reductions in fetuin-A following aerobic exercise training have been attributable to decreases in waist circumference and body weight and increases in adiponectin, an insulin sensitizing hormone (Zhang et al., 2018). Additionally, favorable changes in fetuin-A, liver fat and insulin sensitivity have also been reported following long-term (12 weeks) aerobic and resistance exercise training (Lee et al., 2017). A recent meta-analysis reported that both aerobic and resistance training significantly reduced fetuin-A in dysglycemic and overweight/obese individuals when performed at a moderate or vigorous intensity, with a volume of 60 min per session and a minimum frequency of 4–7 sessions per week (Ramírez-Vélez et al., 2019).

In this study, we found that circulating concentrations of fetuin-A and insulin sensitivity were not significantly affected by RJT performed during HDT bed rest. This may be due to the type, intensity, and duration of the exercise protocol, which was primarily designed to preserve muscle mass and function. In addition, the physical inactivity of bed rest is the primary intervention and the exercise countermeasure in this case is designed to maintain rather than enhance physiological function. We observed a significant reduction in whole body insulin sensitivity, concomitant with an increase in circulating TG after 60 days HDT bed rest in energy-balanced conditions. These findings are in agreement with other bed rest studies (Bergouignan et al., 2009; Rudwill et al., 2015; Kenny et al., 2017), which support links between the decrease in muscle contraction and elevated TG (Bergouignan et al., 2011), decreases in the amount and activity of key proteins associated with muscle glucose uptake (Biensø et al., 2012), and altered mitochondrial function (Kenny et al., 2017). The observation that whole body and liver insulin sensitivity improved in a subgroup of bed rest participants is intriguing and supports the current emphasis on personalized medicine approaches to disease treatment. Interestingly, the improvement in insulin sensitivity was greater in the CTRL group than in the JUMP group. It has previously been highlighted that there is substantial inter-individual variability in physiological responses following lifestyle interventions (Bouchard and Rankinen, 2001; Solomon, 2018; O’Donoghue et al., 2019). In addition, the genes and pathways underlying the response to exercise training and physical inactivity differ (Booth et al., 2012; Pillon et al., 2020). Therefore, it is important to learn more about the possible mediators of the variation in insulin sensitivity following bed rest.

**Figure 3:**
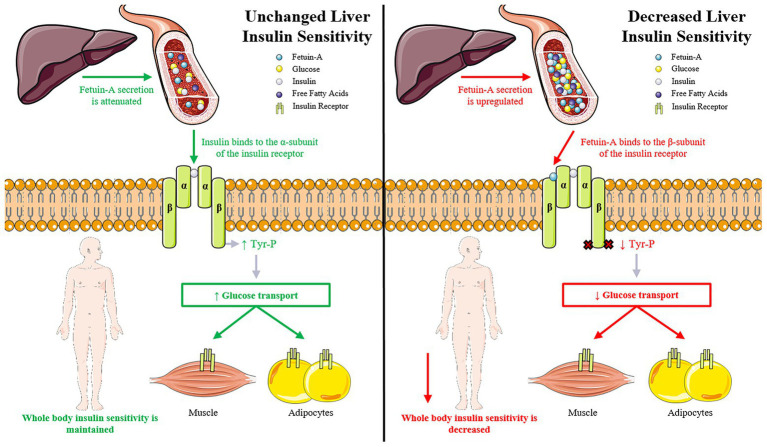
The impact of liver insulin sensitivity and fetuin-A secretion on whole body insulin sensitivity. **(A)** Unchanged liver insulin sensitivity: if liver insulin sensitivity is unchanged then the release of fetuin-A from the liver is attenuated. This allows insulin to bind to the α-subunit of the insulin receptor, which promotes the auto-phosphorylation of the insulin receptor and subsequent tyrosine phosphorylation of the insulin receptor substrate (IRS) proteins. These actions initiate a cascade of events leading to increased glucose uptake in peripheral tissues and an improvement in whole body insulin sensitivity. **(B)** Decreased liver insulin sensitivity: when liver insulin sensitivity is decreased, the release of fetuin-A from the liver is upregulated. Fetuin-A binds to the extracellular portion of the β-subunit of the insulin receptor. Fetuin-A inhibits insulin receptor auto-phosphorylation and tyrosine kinase activity leading to decreased glucose uptake in peripheral tissues and a reduction in whole body insulin sensitivity.

Our analysis identified six participants (26%) that improved insulin sensitivity following HDT bed rest. In addition, there were no significant changes in fetuin-A, fasting glucose, or TG in this subgroup. One possibility is that the improvement in whole body insulin sensitivity may be an indirect effect of liver metabolism. The amount of intra-hepatic lipid (IHL) is strongly linked to liver and whole body insulin resistance (Mu et al., 2018; Trouwborst et al., 2018). If liver insulin sensitivity does not decrease, it is possible that the release of fetuin-A would be attenuated and the negative effect on peripheral tissues would be mitigated (Figure 3). In support of this hypothesis, fetuin-A levels were found to be significantly higher in metabolically-unhealthy compared with metabolically-healthy obese subjects (Khadir et al., 2018). Fetuin-A knockout (KO) mice display enhanced insulin sensitivity, improved glucose tolerance, and lowered plasma lipid content (Mathews et al., 2002). When fed a high fat diet, fetuin-A KO mice remain insulin-sensitive, are resistant to weight gain and have less adiposity than wild-type controls. As there is inter-individual variation in IHL following lifestyle interventions (Winn et al., 2018), it is possible that fetuin-A could be a driver of peripheral insulin resistance and a biomarker to track the physiological responsiveness to bed rest and physical inactivity.

Despite the highly controlled nature of this bed rest study, there are some limitations to acknowledge. Firstly, the sample size for parallel-designed bed rest studies is generally low, in the order of 8–12 participants per group (Bergouignan et al., 2006, 2009; Rudwill et al., 2015, 2018; Kenny et al., 2017). It is possible that the small sample size in the subgroup with improved insulin sensitivity is too small for a definite conclusion, and therefore, we suggest that further studies are required to investigate the potential role of fetuin-A in the regulation of whole body insulin sensitivity following HDT bed rest. Secondly, although it was not the objective of this study, we did not obtain any measurements of liver fat precluding our ability to fully explore the association between fetuin-A and hepatic insulin resistance in response to physical inactivity; this requires further investigation. Thirdly, an OGTT was used to estimate insulin sensitivity. While a euglycaemic-clamp is the gold standard measure, it was not possible in the current study. However, the OGTT sufficiently reflects changes in glucose tolerance and the measurement of whole body insulin sensitivity using the OGTT has been validated previously in healthy non-overweight adults (Trikudanathan et al., 2013). Finally, the results of this study have been obtained from healthy, lean adult males and similar investigations will need to be extended to other populations (e.g., women, elderly, and metabolically unhealthy) to determine the specific links with disease etiology.

In conclusion, we report that 60 days of HDT bed rest led to a significant increase in circulating fetuin-A and decreased insulin sensitivity, which could not be ameliorated by RJT. Exploring individual responses to lifestyle interventions is a growing area of interest in personalized medicine. While HDT bed rest reduced insulin sensitivity at the group level, there was considerable individual responses, including a subgroup for which insulin sensitivity improved. We propose that the regulation of insulin sensitivity is related to circulating fetuin-A, which is attenuated when liver metabolism is maintained. Collectively, our results show that fetuin-A is a candidate biomarker to assess the physiological responses to bed rest and physical inactivity.

## Data Availability Statement

The raw data supporting the conclusions of this article will be made available by the authors, without undue reservation.

## Ethics Statement

The studies involving human participants were reviewed and approved by the North Rhine Medical Association (Ärztekammer Nordrhein) in Düsseldorf, Germany, as well as the Federal Office for Radiation Protection (Bundesamt für Strahlenschutz). The patients/participants provided their written informed consent to participate in this study.

## Author Contributions

KW, DC, and DO’G had full access to the data used in this study and take responsibility for the integrity of the data and the accuracy of the data analysis. The study was designed by KW, DC, DO’G, EM, and PF-M. EM was the project scientist and PF-M leads the work package “biological samples” and “nutrition” for the ESA RSL study. KW, DC, and DO’G conducted the biological sample analysis and were responsible for the formal analysis and interpretation of the data. KW, DC, and DO’G drafted the original manuscript and all authors were involved in the critical review for important intellectual content. Approval of the final manuscript was given by all authors. All authors contributed to the article and approved the submitted version.

### Conflict of Interest

The authors declare that the research was conducted in the absence of any commercial or financial relationships that could be construed as a potential conflict of interest.

## Abbreviations

Adipose IR: Adipose tissue insulin resistance
AUCG: Area under the curve for glucose
AUCI: Area under the curve for insulin
BDC: Baseline data collection
BMI: Body mass index
CTRL: Control group
ELISA: Enzyme-linked immunosorbent assay
HDT: Head-down-tilt
IS: Insulin sensitivity
JUMP: Jump countermeasure group
Liver IS: Liver insulin sensitivity
Muscle IS: Muscle insulin sensitivity
OGTT: Oral glucose tolerance test
RJT: Reactive jump training
PV: Plasma volume
T2DM: Type 2 diabetes mellitus
$\dot{\text{V}}\text{O}{}_{2\,\text{p}\,\text{e}\,\text{a}\,\text{k}}$: Peak aerobic capacity.

## References

[ref1] Abdul-Ghani M. A. Matsuda M. Balas B. DeFronzo R. A. (2007). Muscle and liver insulin resistance indexes derived from the oral glucose tolerance test. Diabetes Care 30, 89–94. 10.2337/dc06-1519, PMID: 17192339

[ref2] Ade C. J. Broxterman R. M. Barstow T. J. (2015). VO(2max) and microgravity exposure: convective versus diffusive O(2) transport. Med. Sci. Sports Exerc. 47, 1351–1361. 10.1249/MSS.0000000000000557, PMID: 25380479

[ref3] Alis R. Sanchis-Gomar F. Lippi G. Roamgnoli M. (2016). Microcentrifuge or automated hematological analyzer to assess hematocrit in exercise? Effect on plasma volume loss calculations. J. Lab. Autom. 21, 470–477. 10.1177/2211068215577571, PMID: 25795010

[ref4] Bergouignan A. Rudwill F. Simon C. Blanc S. (2011). Physical inactivity as the culprit of metabolic inflexibility: evidence from bed-rest studies. J. Appl. Physiol. 111, 1201–1210. 10.1152/japplphysiol.00698.2011, PMID: 21836047

[ref5] Bergouignan A. Schoeller D. A. Normand S. Gauquelin-Koch G. Laville M. Shriver T.. (2006). Effect of physical inactivity on the oxidation of saturated and monounsaturated dietary fatty acids: results of a randomized trial. PLoS Clin Trials 1:e27. 10.1371/journal.pctr.0010027, PMID: 17016547 PMC1584255

[ref6] Bergouignan A. Trudel G. Simon C. Chopard A. Schoeller D. A. Momken I.. (2009). Physical inactivity differentially alters dietary oleate and palmitate trafficking. Diabetes 58, 367–376. 10.2337/db08-0263, PMID: 19017764 PMC2628610

[ref7] Biensø R. S. Ringholm S. Kiilerich K. Aachmann-Andersen N. J. Krogh-Madsen R. Guerra B.. (2012). GLUT4 and glycogen synthase are key players in bed rest-induced insulin resistance. Diabetes 61, 1090–1099. 10.2337/db11-0884, PMID: 22403297 PMC3331744

[ref8] Blottner D. Hastermann M. Weber R. Lenz R. Gambara G. Limper U.. (2019). Reactive jumps preserve skeletal muscle structure, phenotype, and myofiber oxidative capacity in bed rest. Front. Physiol. 10:1527. 10.3389/fphys.2019.01527, PMID: 32009969 PMC6974579

[ref9] Booth F. W. Roberts C. K. Laye M. J. (2012). Lack of exercise is a major cause of chronic diseases. Compr. Physiol. 2, 1143–1211. 10.1002/cphy.c110025, PMID: 23798298 PMC4241367

[ref10] Bouchard C. Rankinen T. (2001). Individual differences in response to regular physical activity. Med. Sci. Sports Exerc. 33, S446–S451. 10.1097/00005768-200106001-00013, PMID: 11427769

[ref11] Bourebaba L. Marycz K. (2019). Pathophysiological implication of fetuin-a glycoprotein in the development of metabolic disorders: a concise review. J. Clin. Med. 8:2033. 10.3390/jcm8122033, PMID: 31766373 PMC6947209

[ref12] Choi K. M. (2016). The impact of organokines on insulin resistance, inflammation, and atherosclerosis. Endocrinol. Metab. 31, 1–6. 10.3803/EnM.2016.31.1.1, PMID: 26996418 PMC4803543

[ref13] Cox-York K. A. Pereira R. I. (2020). “Biomarkers of insulin resistance” in Insulin resistance: childhood precursors of adult disease. eds. Zeitler P. S. Nadeau K. J. (Cham, Switzerland: Humana Press), 169–193.

[ref14] Dill D. B. Costill D. L. (1974). Calculation of percentage changes in volumes of blood, plasma, and red cells in dehydration. J. Appl. Physiol. 37, 247–248. 10.1152/jappl.1974.37.2.247, PMID: 4850854

[ref15] Ennequin G. Sirvent P. Whitham M. (2019). Role of exercise-induced hepatokines in metabolic disorders. Am. J. Physiol. Endocrinol. Metab. 317, E11–E24. 10.1152/ajpendo.00433.2018, PMID: 30964704

[ref16] Goustin A. S. Derar N. Abou-Samra A. B. (2013). Ahsg-fetuin blocks the metabolic arm of insulin action through its interaction with the 95-kD β-subunit of the insulin receptor. Cell. Signal. 25, 981–988. 10.1016/j.cellsig.2012.12.011, PMID: 23314177

[ref17] Gratas-Delamarche A. Derbré F. Vincent S. Cillard J. (2014). Physical inactivity, insulin resistance, and the oxidative-inflammatory loop. Free Radic. Res. 48, 93–108. 10.3109/10715762.2013.847528, PMID: 24060092

[ref18] Hart D. A. Zernicke R. F. (2020). Optimal human functioning requires exercise across the lifespan: mobility in a 1g environment is intrinsic to the integrity of multiple biological systems. Front. Physiol. 11:156. 10.3389/fphys.2020.00156, PMID: 32174843 PMC7056746

[ref19] Haukeland J. W. Dahl T. B. Yndestad A. Gladhaug I. P. Løberg E. M. Haaland J. W.. (2012). Fetuin A in nonalcoholic fatty liver disease: *in vivo* and in vitro studies. Eur. J. Endocrinol. 166, 503–510. 10.1530/EJE-11-0864, PMID: 22170794

[ref20] Hennige A. M. Staiger H. Wicke C. Machicao F. Fritsche A. Häring H. U.. (2008). Fetuin-A induces cytokine expression and suppresses adiponectin production. PLoS One 3:e1765. 10.1371/journal.pone.0001765, PMID: 18335040 PMC2258416

[ref21] Iroz A. Couty J. P. Postic C. (2015). Hepatokines: unlocking the multi-organ network in metabolic diseases. Diabetologia 58, 1699–1703. 10.1007/s00125-015-3634-4, PMID: 26032022

[ref22] Kenny H. C. Rudwill F. Breen L. Salanova M. Blottner D. Heise T.. (2017). Bed rest and resistive vibration exercise unveil novel links between skeletal muscle mitochondrial function and insulin resistance. Diabetologia 60, 1491–1501. 10.1007/s00125-017-4298-z, PMID: 28500394

[ref23] Khadir A. Kavalakatt S. Madhu D. Hammad M. Devarajan S. Tuomilehto J.. (2018). Fetuin-A levels are increased in the adipose tissue of diabetic obese humans but not in circulation. Lipids Health Dis. 17:291. 10.1186/s12944-018-0919-x, PMID: 30579336 PMC6303986

[ref24] Konda N. N. Karri R. S. Winnard A. Nasser M. Evetts S. Boudreau E.. (2019). A comparison of exercise interventions from bed rest studies for the prevention of musculoskeletal loss. NPJ Microgravity 5:12. 10.1038/s41526-019-0073-4, PMID: 31098391 PMC6506471

[ref25] Kramer A. Gollhofer A. Armbrecht G. Felsenberg D. Gruber M. (2017a). How to prevent the detrimental effects of two months of bed-rest on muscle, bone and cardiovascular system: an RCT. Sci. Rep. 7:13177. 10.1038/s41598-017-13659-8, PMID: 29030644 PMC5640633

[ref26] Kramer A. Kümmel J. Mulder E. Gollhofer A. Frings-Meuthen P. Gruber M. (2017b). High-intensity jump training is tolerated during 60 days of bed rest and is very effective in preserving leg power and lean body mass: an overview of the cologne RSL study. PLoS One 12:e0169793. 10.1371/journal.pone.0169793, PMID: 28081223 PMC5231329

[ref27] Lee S. Norheim F. Gulseth H. L. Langleite T. M. Kolnes K. J. Tangen D. S.. (2017). Interaction between plasma fetuin-A and free fatty acids predicts changes in insulin sensitivity in response to long-term exercise. Phys. Rep. 5:e13183. 10.14814/phy2.13183, PMID: 28270597 PMC5350184

[ref28] Lomonaco R. Ortiz-Lopez C. Orsak B. Webb A. Hardies J. Darland C.. (2012). Effect of adipose tissue insulin resistance on metabolic parameters and liver histology in obese patients with nonalcoholic fatty liver disease. Hepatology 55, 1389–1397. 10.1002/hep.25539, PMID: 22183689

[ref29] Malin S. K. del Rincon J. P. Huang H. Kirwan J. P. (2014). Exercise-induced lowering of fetuin-A may increase hepatic insulin sensitivity. Med. Sci. Sports Exerc. 46, 2085–2090. 10.1249/MSS.0000000000000338, PMID: 24637346 PMC4640446

[ref30] Malin S. K. Mulya A. Fealy C. E. Haus J. M. Pagadala M. R. Scelsi A. R.. (2013). Fetuin-A is linked to improved glucose tolerance after short-term exercise training in nonalcoholic fatty liver disease. J. Appl. Physiol. 115, 988–994. 10.1152/japplphysiol.00237.2013, PMID: 23928114 PMC3798818

[ref31] Mathews S. T. Singh G. P. Ranalletta M. Cintron V. J. Qiang X. Goustin A. S.. (2002). Improved insulin sensitivity and resistance to weight gain in mice null for the Ahsg gene. Diabetes 51, 2450–2458. 10.2337/diabetes.51.8.2450, PMID: 12145157

[ref32] Matsuda M. DeFronzo R. A. (1999). Insulin sensitivity indices obtained from oral glucose tolerance testing: comparison with the euglycemic insulin clamp. Diabetes Care 22, 1462–1470. 10.2337/diacare.22.9.1462, PMID: 10480510

[ref33] Mika A. Macaluso F. Barone R. Di Felice V. Sledzinski T. (2019). Effect of exercise on fatty acid metabolism and adipokine secretion in adipose tissue. Front. Physiol. 10:26. 10.3389/fphys.2019.00026, PMID: 30745881 PMC6360148

[ref34] Mu W. Cheng X. F. Liu Y. Lv Q. Z. Liu G. L. Zhang J. G.. (2018). Potential nexus of non-alcoholic fatty liver disease and type 2 diabetes mellitus: insulin resistance between hepatic and peripheral tissues. Front. Pharmacol. 9:1566. 10.3389/fphar.2018.01566, PMID: 30692925 PMC6339917

[ref35] Narici M. V. de Boer M. D. (2011). Disuse of the musculo-skeletal system in space and on earth. Eur. J. Appl. Physiol. 111, 403–420. 10.1007/s00421-010-1556-x, PMID: 20617334

[ref36] Ochieng J. Nangami G. Sakwe A. Moye C. Alvarez J. Whalen D.. (2018). Impact of Fetuin-A (AHSG) on tumor progression and type 2 diabetes. Int. J. Mol. Sci. 19:2211. 10.3390/ijms19082211, PMID: 30060600 PMC6121429

[ref37] O’Donoghue G. Kennedy A. Andersen G. S. Carr B. Cleary S. Durkan E.. (2019). Phenotypic responses to a lifestyle intervention do not account for inter-individual variability in glucose tolerance for individuals at high risk of type 2 diabetes. Front. Physiol. 10:317. 10.3389/fphys.2019.00317, PMID: 30971951 PMC6443958

[ref38] Pal D. Dasgupta S. Kundu R. Maitra S. Das G. Mukhopadhyay S.. (2012). Fetuin-A acts as an endogenous ligand of TLR4 to promote lipid-induced insulin resistance. Nat. Med. 18, 1279–1285. 10.1038/nm.2851, PMID: 22842477

[ref39] Pedersen B. K. Febbraio M. A. (2012). Muscles, exercise and obesity: skeletal muscle as a secretory organ. Nat. Rev. Endocrinol. 8, 457–465. 10.1038/nrendo.2012.49, PMID: 22473333

[ref40] Peter A. Kovarova M. Staiger H. Machann J. Schick F. Königsrainer A. I.. (2018). The hepatokines fetuin-A and fetuin-B are upregulated in the state of hepatic steatosis and may differently impact on glucose homeostasis in humans. Am. J. Physiol. Endocrinol. Metab. 314, E266–E273. 10.1152/ajpendo.00262.2017, PMID: 29138227

[ref41] Pillon N. J. Gabriel B. M. Dollet L. Smith J. A. B. Sardón Puig L. Botella J.. (2020). Transcriptomic profiling of skeletal muscle adaptations to exercise and inactivity. Nat. Commun. 11:470. 10.1038/s41467-019-13869-w, PMID: 31980607 PMC6981202

[ref42] Priest C. Tontonoz P. (2019). Inter-organ cross-talk in metabolic syndrome. Nat. Metab. 1, 1177–1188. 10.1038/s42255-019-0145-5, PMID: 32694672

[ref43] Ramírez-Vélez R. García-Hermoso A. Hackney A. C. Izquierdo M. (2019). Effects of exercise training on Fetuin-a in obese, type 2 diabetes and cardiovascular disease in adults and elderly: a systematic review and meta-analysis. Lipids Health Dis. 18:23. 10.1186/s12944-019-0962-2, PMID: 30670052 PMC6343360

[ref44] Rudwill F. Bergouignan A. Gastebois C. Gauquelin-Koch G. Lefai E. Blanc S.. (2015). Effect of enforced physical inactivity induced by 60-day of bed rest on hepatic markers of NAFLD in healthy normal-weight women. Liver Int. 35, 1700–1706. 10.1111/liv.12743, PMID: 25413107

[ref45] Rudwill F. O’Gorman D. Lefai E. Chery I. Zahariev A. Normand S.. (2018). Metabolic inflexibility is an early marker of bed-rest-induced glucose intolerance even when fat mass is stable. J. Clin. Endocrinol. Metab. 103, 1910–1920. 10.1210/jc.2017-02267, PMID: 29546280 PMC7263792

[ref46] Solomon T. P. J. (2018). Sources of inter-individual variability in the therapeutic response of blood glucose control to exercise in type 2 diabetes: going beyond exercise dose. Front. Physiol. 9:896. 10.3389/fphys.2018.00896, PMID: 30061841 PMC6055062

[ref47] Stefan N. Häring H. U. (2013). The role of hepatokines in metabolism. Nat. Rev. Endocrinol. 9, 144–152. 10.1038/nrendo.2012.258, PMID: 23337953

[ref48] Stefan N. Hennige A. M. Staiger H. Machann J. Schick F. Kröber S. M.. (2006). α2-heremans-schmid glycoprotein/fetuin-A is associated with insulin resistance and fat accumulation in the liver in humans. Diabetes Care 29, 853–857. 10.2337/diacare.29.04.06.dc05-1938, PMID: 16567827

[ref49] Trikudanathan S. Raji A. Chamarthi B. Seely E. W. Simonson D. C. (2013). Comparison of insulin sensitivity measures in South Asians. Metabolism 62, 1448–1454. 10.1016/j.metabol.2013.05.016, PMID: 23906497 PMC3889665

[ref50] Trouwborst I. Bowser S. M. Goossens G. H. Blaak E. E. (2018). Ectopic fat accumulation in distinct insulin resistant phenotypes; targets for personalized nutritional interventions. Front. Nutr. 5:77. 10.3389/fnut.2018.00077, PMID: 30234122 PMC6131567

[ref51] Unnikrishnan A. G. (2004). Tissue-specific insulin resistance. Postgrad. Med. J. 80:435. 10.1136/pgmj.2004.023176 15299149 PMC1743074

[ref52] Vico L. Hargens A. (2018). Skeletal changes during and after spaceflight. Nat. Rev. Rheumatol. 14, 229–245. 10.1038/nrrheum.2018.37, PMID: 29559713

[ref53] Winn N. C. Liu Y. Rector R. S. Parks E. J. Ibdah J. A. Kanaley J. A. (2018). Energy-matched moderate and high intensity exercise training improves nonalcoholic fatty liver disease risk independent of changes in body mass or abdominal adiposity—a randomized trial. Metabolism 78, 128–140. 10.1016/j.metabol.2017.08.012, PMID: 28941598

[ref54] Zhang L. Y. Liu T. Teng Y. Q. Yao X. Y. Zhao T. T. Lin L. Y.. (2018). Effect of a 12-week aerobic exercise training on serum fetuin-a and adipocytokine levels in type 2 diabetes. Exp. Clin. Endocrinol. Diabetes 126, 487–492. 10.1055/s-0043-115904, PMID: 28750433

