# Supplementary material for: Fetuin-A as a Potential Biomarker of Metabolic Variability Following 60 Days of Bed Rest
Source: Front Physiol. 2020 Oct 19;11:573581. doi: 10.3389/fphys.2020.573581 (PMC7604312; doi:10.3389/fphys.2020.573581)
Supplement: Supplementary file 1 [file Table_1.docx]

Supplementary Material

**Adipose Tissue Insulin Resistance (Lomonaco et al. 2012)**

**Adipose IR =** Fasting NEFA (mmol/L) * fasting insulin (µU/mL).

**Liver Insulin Sensitivity (Matsuda and DeFronzo, 1999)**

**Liver IS =** k / fasting glucose (mg/dL) * fasting insulin (µU/mL), where k = 22.5 * 18.

**Matsuda Equation (Matsuda and DeFronzo, 1999)**

**Matsuda =** 10,000 / √ fasting glucose (mg/dL) * fasting insulin (µU/mL) * glucose mean (mg/dL) * insulin mean (µU/mL).

Glucose_mean_ = 15 * glucose0 + 30 * glucose30 + 30 * glucose60 + 30 * glucose90 + 15 * glucose120 / 120 minutes

Insulin_mean_ = 15 * insulin0 + 30 * insulin30 + 30 * insulin60 + 30 * insulin90 + 15 * insulin120 / 120 minutes

**Muscle Insulin Sensitivity (Abdul-Ghani et al. 2007)**

**Muscle IS =** dG / dt (mg/dL) / mean plasma insulin concentration (µU/mL).

dG/dt represents the rate of decline in plasma glucose concentrations and is calculated as the slope of the least square fit to the decline in plasma glucose from peak to nadir.
