# Supplementary material for: Fetuin-A as a Potential Biomarker of Metabolic Variability Following 60 Days of Bed Rest
Source: Front Physiol. 2020 Oct 19;11:573581. doi: 10.3389/fphys.2020.573581 (PMC7604312; doi:10.3389/fphys.2020.573581)
Supplement: Supplementary file 2 [file Table_2.docx]

Supplementary Material

**Supplementary Table 1.** The effects of 60 days HDT bed rest on physical characteristics when subjects were divided into two subgroups based on an increase or decrease in insulin sensitivity post-bed rest.

| Measurement | Improved IS Subgroup  (n = 6) | | p-value | Decreased IS Subgroup  (n = 17) | | p-value |
| --- | --- | --- | --- | --- | --- | --- |
|  | **Pre** | **Post** |  | **Pre** | **Post** |  |
| Age (years) | 30 ± 7 |  |  | 29 ± 6 |  |  |
| Height (cm) | 180 ± 6 |  |  | 181 ± 6 |  |  |
| BMI (kg/m²) | 24.06 ± 1.67 | 22.97 ± 1.65 | **0.002** | 23.37 ± 1.97 | 22.55 ± 1.83 | **<0.001** |
| BW (kg) | 78.30 ± 6.68 | 74.73 ± 5.86 | **0.003** | 76.56 ± 7.51 | 73.90 ± 7.02 | **<0.001** |
| LM (kg) | 59.03 ± 5.83 | 55.72 ± 5.04 | **0.020** | 55.83 ± 5.67 | 53.53 ± 4.60 | **<0.001** |
| FM (kg) | 16.97 ± 4.16 | 16.60 ± 3.93 | 0.254 | 18.51 ± 5.83 | 18.09 ± 5.36 | 0.143 |
| BMC (kg) | 3.19 ± 0.41 | 3.17 ± 0.40 | 0.455 | 3.03 ± 0.32 | 3.03 ± 0.33 | 0.955 |
| Measurement | **Improved IS Subgroup**  **(n = 5)** | | **p-value** | **Decreased IS Subgroup**  **(n = 15)** | | **p-value** |
|  | **Pre** | **Post** |  | **Pre** | **Post** |  |
| V̇O_2peak_ (L/min) | 3.51 ± 0.75 | 2.78 ± 0.63 | 0.233 | 3.61 ± 0.78 | 2.78 ± 0.53 | **<0.001** |
| V̇O_2peak_ (ml/kgLM/min) | 59.62 ± 11.90 | 50.04 ± 12.85 | 0.282 | 64.31 ± 10.21 | 51.91 ± 7.74 | **<0.001** |

Data are presented as mean ± standard deviation (SD). Significant p values < 0.05 are indicated in bold. Anthropometric measurements were taken on BDC-3 and HDT60. V̇O_2peak_ was measured on BDC-8 and R+1. Abbreviations: IS, insulin sensitivity; BMI, body mass index; BW, body weight; LM, lean mass; FM, fat mass; BMC, bone mineral content; V̇O_2peak_, peak aerobic capacity.

**Supplementary Table 2.** The effects of 60 days HDT bed rest on metabolic characteristics when subjects were divided into two subgroups based on an increase or decrease in insulin sensitivity post-bed rest.

| Measurement | Improved IS Subgroup  (n = 6) | | p-value | Decreased IS Subgroup  (n = 17) | | p-value |
| --- | --- | --- | --- | --- | --- | --- |
|  | **Pre** | **Post** |  | **Pre** | **Post** |  |
| Glucose_0_ (mmol/L) | 5.52 ± 0.80 | 4.96 ± 0.47 | 0.058 | 5.15 ± 0.39 | 5.50 ± 0.51 | **0.011** |
| Glucose_120_ (mmol/L) | 7.15 ± 0.89 | 7.17 ± 2.11 | 0.978 | 7.17 ± 1.65 | 8.61 ± 1.70 | **0.005** |
| Insulin_0_ (pmol/L) | 54.11 ± 11.35 | 50.94 ± 12.06 | 0.279 | 49.23 ± 15.91 | 62.78 ± 20.65 | **0.003** |
| Insulin_120_ (pmol/L) | 359.35 ± 68.99 | 357.95 ± 134.68 | 0.984 | 428.44 ± 279.31 | 542.63 ± 309.92 | 0.062 |
| AUCG (mmol/L*min) | 886.73 ± 102.75 | 884.73 ± 157.82 | 0.957 | 906.41 ± 178.31 | 1031.41 ± 122.54 | **0.002** |
| AUCI (pmol/L*min) | 52506.88 ± 14672.98 | 51507.78 ± 14590.59 | 0.477 | 49145.69 ± 20498.88 | 55664.21 ± 16667.60 | **0.027** |
| NEFA (mmol/L) | 0.35 ± 0.09 | 0.38 ± 0.07 | 0.667 | 0.42 ± 0.14 | 0.50 ± 0.19 | 0.137 |
| TG  (mmol/L) | 1.03 ± 0.39 | 1.03 ± 0.30 | 0.969 | 1.02 ± 0.40 | 1.14 ± 0.38 | **0.009** |
| CHOL (mmol/L) | 4.00 ± 0.95 | 3.96 ± 0.90 | 0.887 | 4.14 ± 0.51 | 4.23 ± 0.66 | 0.519 |
| HDL  (mmol/L) | 1.09 ± 0.14 | 0.87 ± 0.10 | **0.003** | 1.13 ± 0.26 | 0.98 ± 0.19 | **0.007** |
| LDL  (mmol/L) | 2.73 ± 1.03 | 2.92 ± 0.89 | 0.440 | 2.75 ± 0.40 | 3.09 ± 0.53 | **0.003** |

Data are presented as mean ± standard deviation (SD). Significant p values < 0.05 are indicated in bold. Metabolic characteristics were measured on BDC-5 and HDT59. Abbreviations: IS, insulin sensitivity; Glucose_0_, fasting glucose; Glucose_120_, glucose concentrations 120 minutes after the glucose load; Insulin_0_, fasting insulin; Insulin_120_, insulin concentrations 120 minutes after the glucose load; AUCG, area under the curve for glucose for 120 minutes; AUCI, area under the curve for insulin for 120 minutes; NEFA, non-esterified fatty acids; TG, triglycerides; CHOL, total cholesterol; HDL, high-density lipoprotein cholesterol; LDL, low-density lipoprotein cholesterol.

Supplementary Table 3. The effects of 60 days HDT bed rest on metabolic characteristics uncorrected for changes in plasma volume.

| Measurement | CTRL (n = 11) | | JUMP (n = 12) | | Statistics | | | |
| --- | --- | --- | --- | --- | --- | --- | --- | --- |
|  | **Pre** | **Post** | **Pre** | **Post** | **Time** | **Int** | **T*Int** |  |
| Glucose_0_ (mmol/L) | 5.18 ± 0.40 | 5.19 ± 0.31 | 5.32 ± 0.64 | 5.60 ± 0.49 | 0.194 | 0.119 | 0.242 |  |
| Glucose_120_ (mmol/L) | 6.95 ± 1.01 | 8.45 ± 1.74 | 7.36 ± 1.82 | 8.12 ± 1.83 | **0.002** | 0.946 | 0.262 |  |
| Insulin­_0_ (pmol/L) | 44.74 ± 13.28 | 50.97 ± 14.93 | 55.46 ± 14.85 | 70.04 ± 23.89 | **0.007** | **0.030** | 0.240 |  |
| Insulin_120_­† (pmol/L) | 347.16 ± 184.12 | 493.00 ± 318.55 | 468.39 ± 281.21 | 507.36 ± 277.21 | **0.032** | 0.545 | 0.305 |  |
| AUCG (mmol/L*min) | 878.09 ± 128.83 | 975.23 ± 125.63 | 922.54 ± 187.30 | 1022.34 ± 120.58 | **<0.001** | 0.422 | 0.952 |  |
| AUCI (pmol/L*min) | 46007.44 ± 20134.55 | 54070.73 ± 18387.67 | 53630.81 ± 17871.10 | 56919.88 ± 17881.48 | **0.004** | 0.495 | 0.193 |  |
| NEFA (mmol/L) | 0.40 ± 0.13 | 0.40 ± 0.13 | 0.41 ± 0.14 | 0.53 ± 0.15 | 0.081 | 0.160 | 0.106 |  |
| TG (mmol/L) | 0.87 ± 0.26 | 1.00 ± 0.26 | 1.16 ± 0.45 | 1.24 ± 0.42 | **0.009** | 0.082 | 0.544 |  |
| CHOL (mmol/L) | 4.09 ± 0.72 | 4.23 ± 0.80 | 4.11 ± 0.57 | 4.15 ± 0.51 | 0.349 | 0.898 | 0.623 |  |
| HDL (mmol/L) | 1.16 ± 0.17 | 0.98 ± 0.14 | 1.08 ± 0.27 | 0.93 ± 0.14 | **<0.001** | 0.384 | 0.574 |  |
| LDL (mmol/L) | 2.76 ± 0.71 | 3.08 ± 0.72 | 2.73 ± 0.51 | 3.07 ± 0.56 | **<0.001** | 0.932 | 0.961 |  |
| Fetuin-A† (g/L) | 0.38 ± 0.16 | 0.46 ± 0.11 | 0.40 ± 0.12 | 0.55 ± 0.22 | **<0.001** | 0.540 | 0.895 |  |
| Matsuda Index† | 5.49 ± 2.21 | 4.27 ± 1.21 | 4.37 ± 1.87 | 3.42 ± 1.23 | **<0.001** | 0.080 | 0.507 |  |
| Liver IS | 0.75 ± 0.30 | 0.64 ± 0.19 | 0.58 ± 0.18 | 0.45 ± 0.15 | **0.012** | **0.025** | 0.842 |  |
| Muscle IS | 0.03 ± 0.04 | 0.01 ± 0.01 | 0.02 ± 0.01 | 0.01 ± 0.01 | 0.051 | 0.365 | 0.300 |  |
| Adipose IR | 2.50 ± 0.99 | 3.01 ± 1.48 | 3.49 ± 2.01 | 5.29 ± 2.20 | **0.002** | **0.021** | 0.065 |  |

Data are presented as mean ± standard deviation (SD). Significant p values < 0.05 are indicated in bold. † denotes that data was transformed for statistical analysis. Metabolic characteristics were measured on BDC-5 and HDT59. Abbreviations: CTRL, control group; JUMP, jumping countermeasure group; Time, main effect of time; Int, main effect of intervention; T*Int, time*intervention interaction effect; Glucose_0_, fasting glucose; Glucose_120_, glucose concentrations 120 minutes after the glucose load; Insulin_0_, fasting insulin; Insulin_120_, insulin concentrations 120 minutes after the glucose load; NEFA, non-esterified fatty acids; TG, triglycerides; CHOL, total cholesterol; HDL, high-density lipoprotein cholesterol; LDL, low-density lipoprotein cholesterol; IS, insulin sensitivity; IR, insulin resistance.
